# Gut microbiome colonization and development in neonatal ruminants: Strategies, prospects, and opportunities

**DOI:** 10.1016/j.aninu.2021.03.004

**Published:** 2021-05-29

**Authors:** Muhammad A. Arshad, Faiz-ul Hassan, Muhammad S. Rehman, Sharon A. Huws, Yanfen Cheng, Ahmad U. Din

**Affiliations:** a Institute of Animal and Dairy Sciences, Faculty of Animal Husbandry, University of Agriculture, Faisalabad, 38040, Pakistan; b Laboratory of Gastrointestinal Microbiology, National Center for International Research on Animal Gut Nutrition, Nanjing Agricultural University, Nanjing, 210095, China; c Key Laboratory of Buffalo Genetics, Breeding and Reproduction Technology, Ministry of Agriculture and Guangxi Buffalo Research Institute, Chinese Academy of Agricultural Sciences, Nanning, 530001, China; d School of Biological Sciences, Institute for Global Food Security, Queen's University of Belfast, Belfast, BT9 5DL, GB-NIR, UK; e Drug Discovery Research Center, Southwest Medical University, Luzhou, 646000, China

**Keywords:** Gut development, Microbial colonization, Rumen microbiota, Health, Performance

## Abstract

Colonization and development of the gut microbiome is a crucial consideration for optimizing the health and performance of livestock animals. This is mainly attributed to the fact that dietary and management practices greatly influence the gut microbiota, subsequently leading to changes in nutrient utilization and immune response. A favorable microbiome can be implanted through dietary or management interventions of livestock animals, especially during early life. In this review, we explore all the possible factors (for example gestation, colostrum, and milk feeding, drinking water, starter feed, inoculation from healthy animals, prebiotics/probiotics, weaning time, essential oil and transgenesis), which can influence rumen microbiome colonization and development. We discuss the advantages and disadvantages of potential strategies used to manipulate gut development and microbial colonization to improve the production and health of newborn calves at an early age when they are most susceptible to enteric disease. Moreover, we provide insights into possible interventions and their potential effects on rumen development and microbiota establishment. Prospects of latest techniques like transgenesis and host genetics have also been discussed regarding their potential role in modulation of rumen microbiome and subsequent effects on gut development and performance in neonatal ruminants.

## 1 Introduction

The gut microbiota is essential for crucial functional activities, such as gut development, feed digestion and utilization, and immune response in livestock animals. Changes in gut microbial colonization in livestock animals during early life often result in permanent effects on the establishment of rumen microbiota and resultant effects on host phenotype (Furman et al., 2020; Guo et al., 2020). Ruminal microbes are fundamentally anaerobic and produce various compounds during rumen fermentation, which are directly used by the host or other microbes. Methane is produced by methanogens through utilizing metabolic hydrogen during rumen fermentation (Hassan et al., 2020). Overall, enteric methane emission from ruminants constitutes about 18% of total methane emissions from all anthropogenic sources (Mizrahi et al., 2021). Methane is the second most potent greenhouse gas after CO_2_ that contributes to global warming. Moreover, methane emanation is a loss of dietary energy, which would otherwise be used by the host to produce meat and milk. Therefore, manipulating the rumen microbiome of livestock animals is considered as an approach to reduce their environmental impacts while increasing production efficiency (O'hara, 2019). During later stages of life, it is more challenging to change rumen microbial ecology over a long time, however, in early life, a favorable microbiome can be implanted via dietary or management interventions with potentially a long-lasting effect (Dill-Mcfarland et al., 2017; Furman et al., 2020; Malmuthuge et al., 2019; Palma-Hidalgo et al., 2020). Gastrointestinal tracts (GIT) of ruminants are colonized by different micro-organisms (bacteria, archaea, virus, protozoa, and fungi) in their adult life, and are considered sterile at birth. However, within 20 min of birth, fibrolytic bacteria and methanogens appear in the rumen of newborn animals (Guzman et al., 2015). One day post-birth the microbial density of rumen microbiota reaches up to 10^9^ cells per milliliter (Li et al., 2012; Yáñez-Ruiz et al., 2015), with cellulolytic bacteria, *Ruminococcus flavefaciens*, *Ruminococcus albus* and members of the *Prevotella* genus often being detected at that time. These microbes are involved in various critical energy-harvesting functions (McLoughlin et al., 2020; Shabat et al., 2016), such as cellulose and hemicellulose degradation (Jami et al., 2013).

The ruminant digestive system switches from that of a monogastric to that of a fully functional foregut rumen fermenter post-weaning, with an ability to digest fibrous feed. During the suckling period of calves, milk bypasses the rumen due to the esophageal groove. The developed rumen comprises 60% to 80% of the total digestive system volume as compared with the monogastric stomach in early life. Besides this, during early life, rumen villi are not yet developed which are necessary for the absorption of nutrients (Krishnamoorthy and Moran, 2012; Van Soest, 2018). Rumen microbial populations have a considerable impact on rumen structure and physiological development. Initial microbial GIT colonizers constitute both aerobic and facultative anaerobic microbial taxa, which later on mostly are replaced by anaerobic taxa (Minato et al., 1992). Consequently, one-day-old calves have a very different bacterial population compared to three-day-old calves (Jami et al., 2013). The oxidative condition within the rumen is a primary regulator of change within the rumen ecosystem and redox in newborns, with an inert impact on the advancement of methanogenic species (Friedman et al., 2017). The primary changes which occur as rumen development ensues include modification in density configuration within the Bacteroidetes phylum. In the developed rumen, this phylum is dominated by the genus *Prevotella* across several species (Henderson et al., 2015). Nevertheless, during primary stages of development, *Bacteroides* is the main genus within phylum Bacteroidetes and is immediately replaced by the *Prevotella* during the first 2 months of life (Rey et al., 2014).

The period from birth to weaning is important for rumen microbial colonization and adaptation. Regarding this, transmission is one of the most important factors that affects the development of microbiota in the GIT (Dominguez-Bello et al., 2010; Francino, 2014). The composition of this complex microbial community is shaped by the highly dynamic physical, chemical, and predatory conditions within the rumen, and potentially by genetic factors of the host (Sasson et al., 2017). Interaction of the host and microbes is essentially responsible for the development of microbial colonization known as co-evolution (Malmuthuge et al., 2015; O'hara et al., 2020a). The microbial population is established by successive waves where convergence of microbial populations is seen reaching a more stable population structure (Furman et al., 2020).

Once the development and maturation of rumen and the microbiome are complete, it is difficult to permanently manipulate or change the rumen ecosystem due to microbial adaptation and resilience to external mediators (Clemmons et al., 2019) and the control that the host genome has been shown to have on the microbiome (Ribeiro et al., 2017; Abbas et al., 2020; Weimer et al., 2017). That is why developing a rumen ecosystem during weaning age is key for getting higher growth rates and better health at a later stage of life (De Barbieri et al., 2015; O'hara et al., 2020b). Another possible strategy for improving feed efficiency is the fortification of rumen microorganisms in calves during early life. The main objective of such strategies is to overcome the risk of undesirable health consequences associated with an altered gut microbiome in neonatal animals and restoration of the gut microbial community following dysbiosis. A complete understanding of early gut colonization is necessary to design different effective strategies to manipulate the GIT microbiome.

**Fig. 1:**
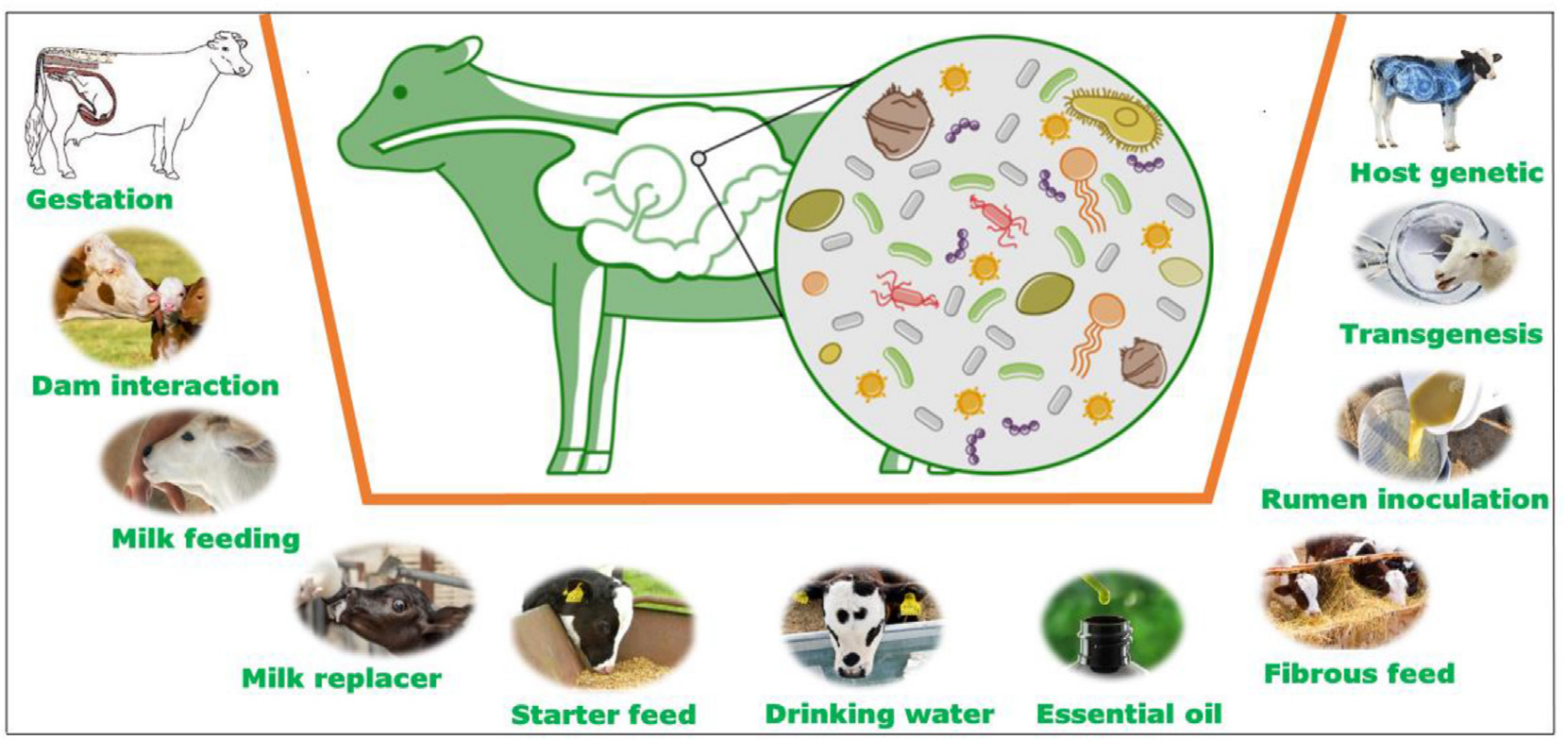
Possible factors involved in rumen microbial colonization during early life.

Although a wealth of literature is available on different aspects of the rumen microbiome in adult animals and early colonization of gut microbiota, information regarding the role of host genetics and microbial interactions in the early development of the gut microbiota is limited. This review focuses on providing insights into the initial colonization of rumen microbiota, while focusing on challenges in our understanding of the complex interaction between different factors, especially between the host and the microbiome. We also present some potential strategies to manipulate the rumen microbiome in early life (Fig. 1), to desirably enhance gut development and microbial colonization for improved health and production in ruminants.

## 2 Relationship of gut microbiota with host immunity

**Fig. 2:**
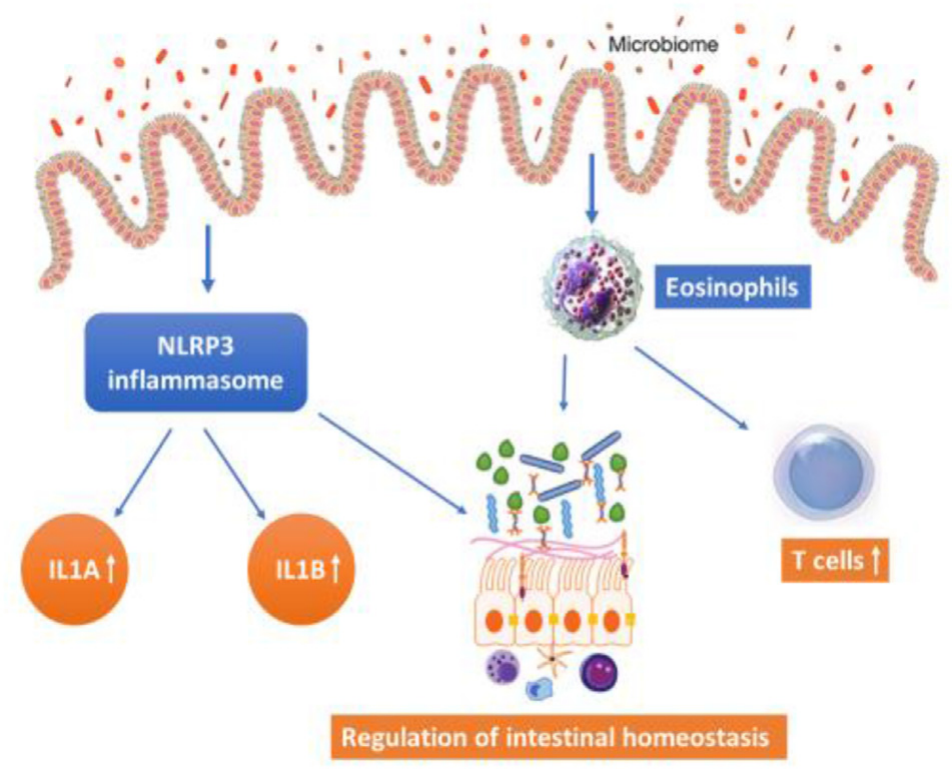
Activation of the NLRP3 inflammasome and the expansion of eosinophils in ileal tissue. NLRP3 inflammasome (member of multi-protein innate immune complex) regulates intestinal homeostasis (Hirota et al., 2011). Interleukin-1 alpha (IL1A) and interleukin-1 beta (IL1β) proteins are mediated through NLRP3 inflammasome. IL1A, known as “pro-inflammatory”, stimulates the activity of genes involved in inflammation and immunity (Lee et al., 2008). IL1β is involved in T-cell activation, antibody production, and promotes Th17 differentiation of T-cells (Tominaga et al., 2000). NLRP3 = NOD-like receptor pyrin domain-containing protein 3.

Developing better immunity is crucial for the prevention and elimination of pathogens, and to maintain the health status of young ones. Newborn ruminants have no immunity at the time of birth, which can be induced through colostrum feeding from the mother. Immunoglobulins (IgG, IgA, and IgM) in colostrum provide immunity and protection against inhaled and ingested pathogens (Woof and Kerr, 2004). The presence of IgA reduces intestinal pro-inflammatory signaling to mediate tolerance in the gut, and regulate gut bacterial composition while maintaining intestinal homeostasis between the host and GIT microbes (Bauer et al., 2006a). Microbial colonization has a critical role in the development of host innate immunity (Janeway Jr and Medzhitov, 2002). Tracheal antimicrobial peptide (TAP) and β-defensin genes are front-line protectants against pathogens (Caverly et al., 2003). TAP also provides a link between innate and adaptive immune responses against microbial invasions (Yang et al., 2002). Notably, the β-defensin 1 and β-defensin 2 were detected in the first 6 to 8 weeks of life, with substantial expression in pre-natal lambs in the digestive tract (Huttner et al., 1998; Meyerholz et al., 2006). Toll-like receptors (TLR) are another class of host proteins with important roles in inducing an immune response in the GIT with the help of gut microbes in calves (Malmuthuge et al., 2012). Exogenous bacterial colonization in calves triggers immune and defense responses causing induction of expression of different genes involved in the adaptive and innate immune system (Li et al., 2019) and studies indicate that the genes related to host innate immunity have an important role in developing a composite interface between the colonized microbial community of fresh rumen and host immune surveillance. The majority of studies have reported developmental ontogeny of the digestive tract in the neonatal calf (usually on the rumen), however, the richest site of immune cell deposition along the intestine is in the ileum (small intestine) of cattle. The submucosa of the ileum has lymphoid nodules called Peyer's patches, also known as “immune sensor” of the intestine (Jung et al., 2010). Recently, Lyons et al. (2020) revealed an important portal of host–microbe interaction with the presence of large number of innate immune cells (eosinophils ad macrophages) in the ileum. A strong relationship between the relative abundance of *Bacteroidetes* and T cells has been observed in the calf's ileum. Webb et al. (2016) demonstrated that genus *Bacteroidetes* could stimulate regulatory T cells which promote epithelial repair, promote tolerance to microbes and initiate suppression of immune responses to self and bacterial antigens. Appropriate activation of the inflammasome sensors (such as NOD-like receptor pyrin domain-containing protein 3 [NLRP3]) and the expansion of eosinophils in ileal tissue are likely to be important in terms of regulating ileal homeostasis and maladaptive inflammation immediately after birth and warrants further investigation (Fig. 2).

## 3 Factors affecting the development of gut microbiota in neonatal ruminants

The colonization and establishment of microbiota play a key role in the development and function of the GIT, which is subsequently associated with higher body weight and feed efficiency of young ruminants (Wickramasinghe et al., 2020). There are numerous factors that directly or indirectly affect microbial colonization and gut development in ruminants. Some crucial considerations regarding these are discussed below.

### 3.1 Gestation

Maternal nutrition in pregnancy has an important role in the health of the offspring later in life through modulating *in utero* developmental processes. In this regard, gut microbiota provides a potential mediating factor (Chu et al., 2016). Transmission of bacteria from the mother to her offspring is required for developing a healthy nascent microbiome, which may impact growth, immune system maturation, and even neurodevelopment of human infants (Foster and Neufeld, 2013; Hansen et al., 2012; Schwarzer et al., 2016). Similarly, microbial colonization in the bovine gut in early life is important for efficient metabolic functions, immune system, and future health (De Agüero et al., 2016). However, microbes were observed in newborn calf meconium (Alipour et al., 2018), suggesting that microbial colonization of the bovine hindgut might begin before birth, and that prenatal dam-to-fetus efflux of commensal bacteria occurs *in utero* via placental barrier transmission (Funkhouser and Bordenstein, 2013). However, Malmuthuge and Griebel (2018) reported that the fetal intestine and fetal environment are sterile during the third trimester of pregnancy and after the rupture of the amniotic membrane, colonization of the GIT only begins during the birth process. However, another study has also supported the idea of dam-to-fetus efflux of bacteria by detecting hindgut bacteria in neonatal calves immediately after birth (Elolimy et al., 2019). Therefore, there is currently no consensus on whether or not colonization occurs pre-birth. It remains unclear whether small numbers of microbial cells also translocate to the fetus through the placenta (Seferovic et al., 2019; Theis et al., 2019).

During pregnancy, antimicrobial treatments may increase the risk of immunological and metabolic disorders in offspring (Korpela et al., 2016). Maternal microbiota plays a key role in the development of the intestinal immune system during the fetal period through circulating microbial metabolites (De Agüero et al., 2016). Dietary supplementation of methionine to maternal diet during late pregnancy in dairy cows has exhibited a positive impact on hindgut functionality and health in their offspring via modifications in the fecal microbiota and metabolome although feed intake was similar (Elolimy et al., 2019). Furthermore, these modifications may also provide essential nutrients to the neonate and prevent pathogen colonization of the hindgut (Elolimy et al., 2020). Other beneficial effects of the maternal supply of methionine to neonatal calves, including faster maturation of hepatic metabolic pathways and better innate immune function have also been observed (Jacometo et al., 2017; Wang et al., 2019a).

### 3.2 The microbiota of newborn calves and their dams

Bacterial colonization in the GIT of newborns depends upon microbes possessed by the mother and the environment, during fetal development and after birth (Maynard et al., 2012; Stinson et al., 2017), but the observations are controversial due to challenges in reliable sampling and analysis of low-abundance microbial taxa (Alipour et al., 2018). Irrespective, on the whole, exposure of an offspring to the mother's microbial environment appears to be beneficial in terms of life-long immune homeostasis (Torow and Hornef, 2017). The fecal microbiota of newborn calves was derived from the inoculation of the birth canal during birth, because the cow vaginal and calf fecal microbiota are quite similar (Klein-Jöbstl et al., 2019). However, other sources were not examined, which is why the environment and the uterus *ante natum* might be a source of colonization. The newborn rectal microbiota in farm animals usually resembles the dam's oral rather than fecal or vaginal vestibular microbiota; this is mainly composed of Firmicutes*,* Proteobacteria, Actinobacteria, and Bacteroidetes. Following birth in lambs, before colostrum feeding, the most abundant genera included: *Actinobacillus* (6.1%), *Halomonas* (5.7%), *Mannheimia* (3.8%), *Sphingomonas* (3.8%), and *Lactobacillus* (1.3%) (Wang et al., 2019b). The possible sources of these bacteria are the mother's vagina, skin, and the environment (Dominguez-Bello et al., 2010; Pannaraj et al., 2017).

Recently, metagenomic analysis of fecal samples of pre-weaned calves and lactating cows (from 17 dairy farms) revealed an abundance of Bacteroidetes and Verrumicrobia in calves, while Firmicutes, Spirochaetes, Deinococcusthermus, Lentisphaerae, Planctomycetes, and Chlorofexi dominated in cows (Haley et al., 2020). This indicates that composite feces from pre-weaned calves (up to 8 weeks of age) harbor different bacterial communities than lactating cows. Recently, Schwaiger et al. (2020) reported a significant increase in the abundance of *Enterococcus* in the intestine of newborn healthy calves between 6 and 24 h of birth. The relative abundance of *Lactobacillus* was also increased in the intestinal tract of calves during the first 7 d after birth (Takino et al., 2017).

There is a perception that vertical transmission of maternal microbiota has a role in microbial colonization of neonatal GIT (Gritz and Bhandari, 2015). Recently, Guzman et al. (2020) demonstrated that fetal GIT was spatially colonized before birth by a pioneer microbiome. They showed that relative abundance of bacterial and archaeal communities in calf fetal fluids and tissues changed between 5 and 7 months of gestation. These findings indicate that initial colonization of the GIT can occur before 5 months gestation, which suggests that microbial colonization of the fetus is a developmental process. However, there is a need to evaluate the path of initial colonization of these microbiomes, their involvement in metabolic interactions with the fetus, and in priming fetal immunity.

The early hindgut microbiome and metabolome at birth before colostrum feeding partly determine feed efficiency (Elolimy et al., 2020). Lambs reared in close contact with their dam exhibited earlier establishment of cellulolytic bacteria during early life compared to lambs with restricted dam exposure (Fonty et al., 1989). Thus, a metabolically active microbiome could be established through the transmission of microbes from the dam to the newborn calf and the colonization of a species-specific microbiome (Belanche et al., 2019). The lambs reared without their dam had no rumen protozoa and exhibited lower microbial diversity, whereas natural rearing accelerated the rumen microbial development and facilitated the transition to a solid diet. Colonization of the alimentary canal probably depends upon the source of connection between newborn calves and their environment. In most cases, feces act as the main source of colonization for the alimentary tract. Piglets that were kept in an environment heavily contaminated with their dam's feces were active at birth and started walking sooner. This favored the rapid entry of considerable numbers of bacteria into the alimentary tract, leading to a massive proliferation of *Escherichia coli*, *Clostridium welchii* and *Streptococci* in the stomach and flooding of the small intestine with these organisms during the short period (Smith, 1965). The incidence of dysbiosis during the neonatal period necessitates potential strategies to restore gut microbiota to avoid adverse health effects associated with the altered gut microbiome. Fecal matter transplantation (FMT) also known as bacteriotherapy, is an intervention that involves the transfer of whole gut microbiome from healthy individuals to microbial dysbiosis individuals (Choi and Cho, 2016). Recently, the use of super-donors to completely restore microbial dysbiosis has been proposed as an option to avoid unreliable and short-term FMT restoration (Wilson et al., 2019). Malmuthuge and Griebel (2019) showed that microbial dysbiosis can be recovered, through reconnecting subsections of the surgically isolated intestinal segment to the adjacent intact intestine during early life in lambs. This model provides insights to explore the effect of early microbial dysbiosis throughout the prolonged neonatal period in large mammals. The intestinal microbiome in vaginally born neonatal calves had a low number of bacterial phyla (Proteobacteria, Firmicutes, Actinobacteria and Bacteroidetes), suggesting a shared microbiota between calf meconium and the maternal vaginal vestibulum (Alipour et al., 2018; Yeoman et al., 2018; Klein-Jöbstl et al., 2019). A recent study showed that meconium of full-term calves delivered by elective caesarean section in most cases harbored a small amount of diverse bacterial DNA and possibly rare culturable bacteria. In the amniotic fluid, bacteria were not observed by 16S qPCR or culturing, but a microbial DNA profile was distinguishable from controls by amplicon sequencing. Based on these findings, it is suggested that calves encounter utero maternal–fetal transmission of bacterial components, but the prenatal acquisition of live bacteria is likely not physiologically significant (Husso et al., 2020).

### 3.3 Colostrum and milk feeding

Diet after birth essentially mediates the colonization of gut microbiota in farm animals. Colostrum feeding is a recommended practice in ruminants to ensure proper early nutrition and development of mucosal immune response. It can effectively prevent the colonization of pathogenic microorganisms in the GIT while decreasing inflammatory reactions (Fischer, 2017; Hang, 2019). Milk can potentially prevent the binding of potential pathogens with gut epithelium as it contains 40 oligosaccharides and lactoferrin which serve as antimicrobial, anti-inflammatory, and iron-chelating agents (Pacheco et al., 2015). After colostrum intake on day 2 of age, an increased *Lactococcus* abundance was observed with low rumen pH (less than 4) in neonatal lambs (Wang et al., 2019a). Feeding colostrum can resist colonization of *Mycobacterium avium* subsp. *Paratuberculosis* in newborn calves infected with these bacterial species (Stabel et al., 2019). Increased milk intake (20% of body weight) after birth has also been shown to result in improved health status (greater blood tumor necrosis factor-α and lower blood urea nitrogen) of Holstein female calves, when compared with low milk intake (10% body weight) during the first 3 weeks of life (Alimirzaei et al., 2020). Increased *Lactobacillus* abundance was observed in the feces of calves fed more milk, which was associated with improved gut environment and reduced inflammation. Recently, Van Keulen et al. (2020) reported that increased milk allowance (20% of body weight) improved villus height, width and surface area of the small intestine in newborn calves. This improvement may be crucial for intestinal integrity and barrier function with increased cell growth and proliferation in newborn calves.

Early colonization by Gram-negative bacteria in the GIT might adversely affect calf health and growth performance of dairy calves (Bach et al., 2017). Colostrum feeding has been shown to stimulate the colonization of *Clostridium* and *Bifidobacterium,* while inhibiting *E. coli* (Song et al., 2019). Time of colostrum feeding after birth also affects the immune status of newborn calves, as delaying colostrum feeding up to 12 h after birth decreased the passive transfer of immunoglobulin G (IgG) in calves (Fischer et al., 2018a). Recently, Ma et al. (2019) reported that delayed feeding of first colostrum to neonatal calves did not affect the relative abundance of ileum mucosa-associated bacteria at the phylum level. Colostrum replacer can be fed successfully as an alternative to maternal colostrum or as a supplement to colostrum with low IgG (Lopez et al., 2020). However, studies have shown that maternal colostrum feeding to neonatal lambs enhanced volatile fatty acids (VFA) production at weaning compared with the colostrum alternative group (Belanche et al., 2019). However, later in life when all lambs were grouped on the same pasture, they developed similar rumen prokaryotic communities. Based on these findings, it has been suggested that dietary interventions in early life can alter the colonization of gut microbiota with desirable consequences (De Barbieri et al., 2015; Zhong et al., 2014). Although, some effects of early life interventions are long-lived, some of the acquired changes are temporary. However, post-weaning programming exhibits substantial effects on rumen microbiota and performance indices (Dill-Mcfarland et al., 2019). Consequently, it is suggested that effective manipulation of the GIT microbiome should focus on the weaning transition period. This is mainly because dietary interventions aimed at or immediately after weaning are likely most effective with long term effects on rumen microbial ecology and functions (Belanche et al., 2019; Dill-Mcfarland et al., 2017). Very limited data is available regarding the genetics and dynamics of microbial communities of milk-fed newborns before the functional development of the rumen. Therefore, further studies are required to better understand the mechanism of gut colonization and the precise role of different factors affecting microbial establishment, especially host genetics and microbial interactions.

Although feeding colostrum promotes weight gain and colonization of beneficial bacteria in the small intestine of newborn calves (Hammon et al., 2002; Malmuthuge et al., 2015) during colostrum handling, poor hygiene practices can cause microbial contamination, ultimately leading to the exposure of young ruminants to pathogens (Morrill et al., 2012). To prevent such contamination, heat treatment of colostrum is usually suggested, which not only inhibits potential pathogens (*Mycoplasma bovis*, *Listeria monocytogenes*, *E. coli,* and *Salmonella* *enteritidis*) in the colostrum but also helps in transferring passive immunity to young ruminants (Godden et al., 2006; Johnson et al., 2007; Rafiei et al., 2019). Intestinal absorption of IgG can be increased through feeding heat-treated colostrum (60 °C for 30 min) owing to a lower bacterial concentration in this colostrum (Gelsinger et al., 2015; Salazar-Acosta and Elizondo-Salazar, 2019; Saldana et al., 2019). Bovine colostrum contains various bioactive proteins such as immunoglobulins, lactoferrin, and lactoperoxidase. Chatterton et al. (2020) subjected bovine colostrum to different processing techniques including low-temperature, long-time pasteurization (63 °C, 30 min) or high-temperature, short-time pasteurization (72 °C, 15 s) and spray-drying (with or without γ-irradiation) to remove microbial contamination. They reported that the high-temperature, short-time pasteurization technique damages several bioactive proteins which are highly sensitive. A recent study showed that heated colostrum improves the abundance of *Bifidobacterium* and reduces *Enterobacteriaceae* and *E. coli* abundance in neonatal calves at 12 h of life (Song et al., 2019). Bovine colostrum contains oligosaccharides, which play a key role in the stimulation of growth of *Bifidobacterium* (Ward et al., 2007). Heat treatment can denature the oligosaccharide-protein bond resulting in free oligosaccharide contents (Neeser et al., 1991). Studies have shown that heated colostrum contains substantially higher oligosaccharides (3,511.6 μg/g) as compared to non-heated colostrum (1,329.9 μg/g) of Holstein cows (Fischer et al., 2018b). Additionally, calves fed heated colostrum have shown increased fold changes of mucosa-associated *Bifidobacterium* and *Clostridium* cluster XIVa in the colon (Song et al., 2019). These data show that heat-treated colostrum can protect the intestine against pathogens, as well as stimulating the development of the immune function. Additionally, chemical additives are also commonly used for preserving colostrum as they also may reduce microbial counts. In a recent study by Morales-Delanuez et al. (2020), glycerol and propylene glycol were used to reduce microbial counts and preserve immune properties in heat-treated goat milk. They reported that glycerol addition to goat colostrum before heat treatment showed no effect on immunogenic properties of colostrum and reduction of microbial (bacterial) counts.

### 3.4 Drinking water

Offering drinking water to newborn dairy calves is restricted up to 17 d of age. Dairy producers generally assume that water contents of milk or milk replacer could fulfill the water requirement of the calves (Usda, 2016). Recently, Wickramasinghe et al. (2019) reported that dairy calves receiving drinking water from the first day post-birth had a higher body weight, fiber digestibility and feed efficiency compared with those that received drinking water at 17 d of age. Early colonization of bacteria in the gut of newborn calves has been observed as a result of offering drinking water from d 1 post birth with increased abundances of *Faecalibacterium prausnitzii* and *Bifidobacterium breve* seen (Wickramasinghe et al., 2020). Furthermore, higher digestibility of acid detergent fiber correlated with *Faecalibacterium*, suggesting that offering drinking water from birth has a positive impact on the ability of the calf to digest a solid diet efficiently even after weaning.

### 3.5 Starter feed

Diet is the main factor that influences the composition of the gut microbiota of neonatal calves (Henderson et al., 2015; Rey et al., 2014). Studies have demonstrated that feeding a solid diet during the pre-weaning period of calves improves performance, microbial establishment rumen development and facilitates weaning transition (Malmuthuge et al., 2019; Yuste et al., 2020). The introduction of a solid diet establishes rumen microbiota as milk bypasses the rumen to enter the abomasum (Heinrichs, 2005). Therefore, managing pre-weaning feeding is important for microbial establishment in the rumen during the weaning period**.** Fermentable carbohydrates have shown most promising effects regarding the composition and activity of the indigenous microbiota of the GIT (Bauer et al., 2006a, 2006b). Supplementing fermentable carbohydrates is essential for the rapid stabilization of the microbial community and bacterial diversity in newly weaned animals (Konstantinov et al., 2003). Therefore, the time of intestinal colonization after birth or during weaning due to rapid change or stress is most crucial for the young animal to consider for optimization for gut development and sustained microbial colonization.

Supplementing starter feed with milk in pre-weaning lambs also promotes rumen development more efficiently and also provides a greater amount of fermentable carbohydrates and ultimately more VFA and acetate proportions to the cecum (Jiao et al., 2015). Increased rumen weight and papillae size, ultimately associated with better health and growth, have been observed in pre-weaning calves with starter feeding (Berends et al., 2012; Pazoki et al., 2017; Sun et al., 2018). Sun et al. (2018) found that starter feeding influenced cecal bacterial communities and decreased inflammatory expression, which has been shown to have a beneficial effect by alleviating the weaning stress in lambs. The relative abundance of the bacterium *Alistipes* was increased in the cecum with increasing age of the lamb fed starter feed and milk. *Alistipes* is associated with fiber degradation in the rumen and utilizes degraded soluble sugars as substrates (Zhang et al., 2017b).

The composition of starter feed is a crucial consideration for effective manipulation of the establishment of gut microbiota and rumen development. This is mainly attributed to the fact that different dominant phyla require specific fermentation substrates in the gut to derive energy required for proliferation and colonization (Li et al., 2012). The starter feed and milk-fed lambs have shown similar phyla prevalence in the cecal mucosa of adult goats (Liu et al., 2017). The dietary supplementation of calves with milk replacer, alfalfa hay, and starter feed has been shown to improve cecal VFA abundance and growth performance compared with maternal grazing and nursing (Wu et al., 2019). Also increased abundance of genera, *Clostridium sensu stricto*, *Escherichia*/*Shigella,* and *Prevotella* was observed in the cecum of calves. These are involved in the utilization of fibrous and non-fibrous carbohydrates and propionate and butyrate production (Zhang et al., 2017a). Early starter feeding has also been shown to influence gene expression in the rumen epithelium as high-grain diets significantly up-regulated the genes involved in VFA absorption and cell proliferation in the rumen (Jing et al., 2018; Yan et al., 2014). Recently, Zhuang et al. (2020) provided new insights into the molecular mechanisms of rumen development in goat kids by revealing the potential role of starter feeding to promote rumen epithelial cell growth, rumen immune function, and activation of lipid metabolism.

### 3.6 Weaning regimen and time

Usually, the term weaning means the reduction of milk allowance and an increasing supply of solid feed to young calves at different ages of life (6 to10 wk). Different feeding programs or time of weaning could affect solid feed intake, rumen development, and digestibility of calves in different ways. Early weaning (4 to 6 weeks) coupled with offering optimum milk allowance (10% of body weight) to calves, encourages early solid feed consumption and early rumen development (Bhatti et al., 2012). Delaying the age of weaning increases body weight gain in calves fed an elevated plane of nutrition before weaning and decreases the transient reduction of weight gain at weaning (Abbas et al., 2017; Meale et al., 2015). An increased solid feed intake has been observed during the transition period when calves were weaned late compared with early-weaned calves (Eckert et al., 2015). Feeding milk to Sahiwal calves (up to 15% of their body weight), coupled with early weaning at 8 weeks of age, saves milk and labor compared to weaning at 12 weeks (Cheema et al., 2016). Similarly, calves weaned at 6 weeks of age have shown greater feed efficiency, weight gain, and higher dry matter and organic matter digestibility compared to late weaned counterparts (Tao et al., 2018). In addition to weaning time, a strategy known as the step-down method has been suggested, in which calves initially receive a high amount of milk, then by increasing water content, milk supply is gradually reduced (Khan et al., 2007a, 2007b). Recently, studies have also followed a step-down method to feed milk replacer up to 7 weeks of age, and reported that providing increased milk quantity during early life encourages solid feed intake, with positive effects on GIT development and growth, as well as calf health (Schäff et al., 2016, 2018).

Weaning is considered as the most critical phase in early life due to the significant dietary changes, which can lead to stress and ultimately disease susceptibility and attenuation of the immune system and growth (Hickey et al., 2003; Schichowski et al., 2010). Improved morphology of the small intestine, organ development, and immune function has been observed in artificially reared lambs weaned at 4 or 6 weeks of age (Mccoard et al., 2020). Furthermore, pre-weaning diet and feeding have shown pronounced and long-lasting impacts on rumen microbial composition due to host-specificity of the rumen microbiome (Abecia et al., 2013, 2014). Calves weaned at 3 weeks of age with the introduction of solid feed intake showed greater microbial abundance in the rumen as compared to the calves weaned at 6 weeks of age (Anderson et al., 1987). Rumen development can be affected by different weaning practices such as milk quantity, weaning age, weaning scheme, and time of introduction of solid feeds to the young ones (Khan et al., 2016). Morphological and physiological development of the rumen can be improved by opting for a suitable weaning strategy such as a step-down weaning method, which has shown a successful weaning of calves at 4 weeks of age (Carballo et al., 2019). Although studies are available regarding the short-term effects of weaning strategies on growth performance and rumen development, further investigations are required for a better understanding of the effects on gastrointestinal function, the rumen microbiome, and calf health.

## 4 Potential strategies to manipulate microbial colonization and gut development

We present potential strategies that can be used to desirably affect the process of initial microbial colonization and gut development in neonatal ruminants.

### 4.1 Rumen transfaunation inoculation from healthy animals

Rumen transfaunation is a process of transferring rumen fluid from healthy animals to others with diverse microorganisms. This transplanted rumen fluid also supplies nutrients and energy to the rumen microbial population (Depeters and George, 2014). Generally, rumen fluid contains different nutrients such as microbial proteins, amino acids, VFA, vitamins, minerals, and diverse enzymes (Elfaki and Abdelatti, 2018; Sarteshnizi et al., 2018). Indigestion of feed in cows could also be eliminated through the transfaunation of small volumes (1 L) of rumen fluid, which also improves rumen function and feed intake of cows (Steiner et al., 2020). Microbial intervention can also be induced artificially in young calves which can potentially affect calf health and establishment of rumen microbiota. Studies have shown that inoculation of fresh or lyophilized or autoclaved rumen fluid from adult animals improved feed efficiency and weight gain of newborn animals (Muscato et al., 2002; Zhong et al., 2014). A recent study in sheep also reported that the inoculation of newborn lambs with mature lyophilized rumen fluid significantly improved starter feed digestibility and growth performance during and after weaning (Yu et al., 2020). Inoculation also improved ruminal propionate concentration, rumen amylase activities, and decreased acetate-to-propionate ratio. The *Streptococcus ruminantium* population was increased with the inoculation of mature lyophilized rumen fluid as it is associated with the utilization of starch (Wang et al., 2019b). Similarly, the relative abundance of 8 bacterial genera (*Acidiphilium, Jeotgalibaca, Polaribacter, Pseudodesulfovibrio, Bdellovibrio, Microbacterium, Eubacterium* and *Sporosarcina*) belonging to 4 phyla (Firmicutes, Proteobacteria, Bacteroidetes and Actinobacteria) were significantly higher in young calves fed with exogenous rumen fluid obtained from an adult cow (Li et al., 2019). A recent study related to goat kids also revealed that inoculation of fresh rumen fluid from adult goat promotes early rumen colonization through the abundant protozoal community, with higher feed intake, greater rumen VFA production and absorption during the preweaning period (Belanche et al., 2020). Inclusion of spray-dried rumen fluid with 1% maltodextrin in suckling dairy calves stimulated the calves’ immune system as revealed by lower serum concentrations of interleukin-6 (Sarteshnizi et al., 2020). These data suggest that providing exogenous rumen fluid to young ruminants not only improves weight gain but also desirably influences diet digestibility, immunity, and health.

### 4.2 Supplementation of prebiotics and probiotics during early life

It is well established that many pathogenic strains, including *E. coli*, cause diarrhea in calves (Moxley and Francis, 1986). These pathogens can be potentially controlled through different feeding strategies. Prebiotics and probiotics have shown desirable effects regarding calf health improvement. Supplementation of prebiotics (galacto oligosaccharides) to 2-week-old calves increased the relative abundance of *Lactobacillus* and *Bifidobacterium* in the colon, which was less pronounced in 4-week old calves (Castro Marquez, 2014). Mannan oligosaccharides (MOS) from yeast cell walls can bind the thread-like fimbriae on pathogenic bacteria and can prevent their intestinal colonization leading to improved gut health (Hooge, 2006; Spring et al., 2015). It has been observed that oligosaccharides as a prebiotic can stimulate beneficial microbiota (Swennen et al., 2006). Slow fermentable polysaccharides might have more potential compared to rapidly fermented oligosaccharides, through the production of short-chain fatty acid and suppression of protein metabolism more distally in the colon. However, supplementing probiotics is a comparatively easier way to manipulate the microbiome during early life, when gut microbiota is being established, because in later stages it is less effective as the rumen is large and highly anaerobic.

Administration of *Bifidobacterium* and *Lactobacillus* to young calves during early life has caused an increase in weight gain and a decrease in the occurrence of diarrhea (Abe et al., 1995; Shehta et al., 2019). Conversely, Vazquez-Mendoza et al. (2019) reported that supplementation of active *Bacillus amyloliquefaciens* in non-medicated milk replacer to calves resulted in similar performance and economic efficiency, compared with ruminants fed pasteurized waste milk. Therefore, replacement of pasteurized waste milk can be possible with non-medicated milk replacers fortified with probiotics without having a negative impact on calf growth rate and health status of calves. For instance, Brown et al. (2005) and Kmicikewycz et al. (2013) demonstrated that feeding non-medicated milk replacer to calves produced higher growth rates than feeding waste milk.

An increase in total serum IgG concentration has been observed following supplementation of *Lactobacillus* in young calves (Al-Saiady, 2010). Similarly, the administration of probiotic *Bifidobacteria* to calves decreased *E. coli* and total coliforms while increasing the relative abundance of *lactobacilli* (Geigerová et al., 2016). Furthermore, they reported that the freeze-dried form of the probiotic is more suitable and convenient for feeding to calves from a practical viewpoint. Supplementation of a fungal probiotic has also been shown to increase dietary intake and live weight gain in weaning calves (Theodorou et al., 1990). Recently, Villot et al. (2019) revealed that yeast (*Saccharomyces cerevisiae boulardii* CNCM I-1079) supplemented to veal calves reduced episodes of diarrhea and maintained a healthy microbial community with *Fecalibacterium* as a predominant genus. Furthermore, fermentation products of *S. cerevisiae* can stimulate the colonization of *Lachnospiraceae* and *Ruminococcaceae* in the rumen and large intestine, respectively (Xiao et al., 2019). The lower relative abundance of *Prevotellaceae* as compared to highly abundant *Lachnospiraceae* has suggested that *S. cerevisiae* might change the fermentation and stimulate the degradation of recalcitrant fiber substrates in the pre-mature rumen of the calves. However, further investigations are required to fully understand the precise role of probiotics and prebiotics in gut microbial colonization and microbiota composition during early life.

### 4.3 Supplementation of plant essential oils during early life

Plant essential oils can change ruminal fermentation and resultant VFA concentrations through altering the ruminal microbial communities (Zhou et al., 2020). Essential oils possess different properties such as antibacterial, antiviral, antifungal, insecticidal, and herbicidal activities, and can induce conformational changes in the cell membrane rendering it less impermeable (Benchaar et al., 2008; Calsamiglia et al., 2007; Miguel, 2010). Different phytochemicals presented in essential oils have different properties; thymol and carvacrol mainly act as potent antimicrobials against pathogens such as *E. coli*, *Salmonella typhimurium*, *Staphylococcus aureus*, *epidermidis* and *L. monocytogenes* (Benchaar et al., 2008). Similarly, oregano essential oil having second-highest oxygen radical scavenging ability after cloves, followed by cinnamon, ginger, and rosemary (Bentayeb et al., 2014). Dietary supplementation of a liquid blend of essential oils and a prebiotic have been shown to improve the immune status of young calves by increasing IgA titers (Swedzinski et al., 2020).

Dietary supplementation of essential oils can also potentially mitigate ammonia nitrogen and methane through deamination and methanogenesis, respectively (Mcintosh et al., 2003). Essential oils have also been shown to improve the intake of calf starter, feed efficiencies, and body weight gain while increasing beneficial bacteria and inhibiting the growth of *E. coli* in the gut (Hill et al., 2007; Santos et al., 2015). Dietary supplementation of an essential oil blend (2.5 g/d) improved the average daily gains and increased immunity of calves compared to calves fed the control and higher inclusion rates (Froehlich et al., 2017). Supplementation of essential oil and phenol improved feed intake and reduced the weaning age of calves (Seifzadeh et al., 2017). Inclusion of oregano essential oil increased the relative abundance of *Prevotella* and *Dialister* bacteria, which indicate its potential to manipulate ruminal fermentation and reduce methane emissions (Zhou et al., 2020).

Similarly, supplementation of ruminant diets with a blend of essential oil and a prebiotic has shown promising effects on growth, feed efficiency, nutrient digestibility, and immunity of calves during a period of 70 d from birth (Liu et al., 2020). Supplementation of essential oil in neonatal calves during the pe-weaning period showed that essential oil can improve the production of short-chain fatty acid and growth of specific rumen bacterial groups. Increased propionate concentrations and ruminal bacterial communities (*Bacteriodetes*) were observed in calves fed essential oil, which was primarily attributed to the higher abundance of *Prevotellaceae* (Poudel et al., 2019). Furthermore, a blend of essential oils (carvacrol, caryophyllene, p-cymene, cineole, terpinene, and thymol) has shown excellent potential to increase propionate concentration and abundance of *Prevotella ruminicola* (Poudel et al., 2018). The above-mentioned findings clearly indicate that essential oils possess the substantial ability to modulate the ruminal ecosystem and microbiota, to improve nutrient utilization, performance, and health during the early stages of the rumen development.

### 4.4 Feeding of fibrous plant material (forage) to stimulate rumen development and microbial colonization

Different feeding regimes may lead to different establishment of microbial populations in the rumen of young ruminants. A meta-analysis of literature spanning from 1998 to 2016 showed that feeding forage in starter feed has the potential to improve feed intake and performance of calves (Imani et al., 2017). Many inconsistent findings regarding dietary intake, weight gain, and growth performance have been reported regarding the provision of forage during the pre-weaning period of the calf (Xiao et al., 2020). However, forage provision has consistently been shown to stabilize rumen pH and enhance the muscular development of the rumen through positively affecting rumen microbiota and fermentation characteristics (Castells et al., 2012; Lin et al., 2018). A recent study showed an increased relative abundance of *Bacteroidales*, *Ruminobacter*, and *Selenomonas* in neonatal lambs after 14 d when they were allowed to graze alongside ewes (Wang et al., 2019a). Forage or pasture feeding in this regard can be a potential source to achieve good muscle or fat deposition, and growth performance of young calves during early life (Khan et al., 2020). On the contrary, feeding a high level of forage to lambs has resulted in low dry matter intake, lower feed efficiency, higher nitrogen excretion, and greater urinary urea-nitrogen loss compared with a low level of forage or solid feed (Yuste et al., 2020). Another study of Xia et al. (2018) reported that feeding a high concentration of forage in mixed diet improved *Lactobacillus* abundance and decreased the abundance of pathogenic microbes, which indicated that a high forage diet for weaned calves improves growth and health of male dairy calves. Furthermore, increased acetate proportion and cellulolytic microbial growth (*R. albus*) in the rumen has been observed as a result of forage provision (Castells et al., 2013; Suárez et al., 2006). Similarly, a higher abundance of *Prevotella* and cellulolytic bacteria (*R*. *flavefaciens* and *R. albus*) was observed in calves supplemented with forages (Kim et al., 2016). Moreover, *Prevotella* was the predominant genus in animals fed high-fiber diets rather than high-caloric diets (Jami et al., 2013). A higher abundance of Bacteroidetes has been observed in the hay supplemented group, with increased rumen growth and volume (Lin et al., 2018). Recently, feeding of a mixture of calf starter and corn silage showed similar growth, weight gain, and health of calves compared to the calf starter (Kehoe et al., 2019). These findings indicate that provision of forage during early life may has a potential impact on the development of predominant microbiota in the rumen.

### 4.5 Transgenesis

Dietary and inoculation techniques are crucial considerations for improving gut development and microbiota colonization in livestock animals to enhance feed efficiency and meet increasing demands for milk and meat while reducing climatic impacts. In this regard, recent biological techniques, such as transgenesis are getting attention for improving the efficiency of animal production and developing alternatives to antibiotics in the future (Wu and Bazer, 2019). Generally, the production performance of livestock depends upon their genome, environmental factors (nutrition and disease etc.), and their interactions (Bazer et al., 2011). Usually, in a transgenic animal, a foreign gene of interest is inserted into its genome. The foreign gene is constructed in vitro using recombinant DNA technology. Generally, transgenic technologies are categorized into two approaches; embryo-mediated and cell-mediated (Laible, 2018). Transgenic animal technology allows the animal to establish a desirable trait. This technology has been used for the successful production of different transgenic animals, including pigs, mice, rats, cattle, rabbits, sheep, and chickens (Prather et al., 2013; Tiley, 2016). Furthermore, Wang et al. (2016), evaluated the effect of a neomycin phosphotransferase transgene by studying changes in the gut microbiota of piglets using high throughput sequencing. Neo-transgenic expression in transgenic pigs not only changed the relative abundance of some bacteria (Firmicutes, Bacteroidetes, and Proteobacteria) in the intestine but also decreased the levels of potentially harmful bacteria such as *Escherichia, Shigella,* and *Hafnia*. From these studies, it is speculated that animal transgenesis is a promising technology in the future to overcome the limitation of conventional methods for improving feed efficiency and improving gut microbiota especially in the early age of life. However, to be used globally, regulations regarding genetically modified organisms would need to be revised.

## 5 Redundancy, resilience, and host specificity of gut microbiota

As mentioned in previous sections, microbial colonization starts at birth, although it has been proposed that it may even start pre-birth, and dynamically proceeds exhibiting substantial changes induced by many factors like birth type, colostrum/milk feeding, diet and antimicrobial treatment (Bokulich et al., 2016). Once microbiota colonizes and occupies specific niches, the relative abundance of the microbial community consists of a core microbiome that is representative of that particular host species which shows inherent resilience to different perturbations like an established ecosystem (Dethlefsen and Relman, 2011; Martínez et al., 2013; Schloissnig et al., 2013). The development of rumen with age significantly affects microbial diversity and colonization (Furman et al., 2020). Rumen bacteria are the most abundant and diverse group of microbes that constitute more than 95% of the total rumen microbiome (Jami EMizrahi, 2012; Petri et al., 2013). The core microbiome in bovines mainly includes Bacteroidetes*,* Firmicutes and Proteobacteria which constitute more than 90% of total rumen bacteria (Petri et al., 2013). Studies have shown that this core microbiome fluctuates within these phyla right after birth but becomes stable after rumen development completes and starts proper functioning. This shows the redundancy and resilience of the core microbiome in bovines and other farm animals. Remarkable similarity observed among functional classes of the microbiome at different early stages of life (age groups) suggests that rumen microbiota possess the effective potential to maintain a sustainable physiological and metabolic potential in ruminants (Jami et al., 2013). Redundancy of rumen microbiome stems from overlapping physiological abilities across multiple microbial taxa (Weimer, 2015).

Studies have shown that relative abundance of core phyla varies distinctly with age, for example, composition at 14 d was different from 42 d in Holstein calves. Although overall 170 genera were observed in calves, the core microbiome just consisted of 45 genera in pre-ruminant calves (Li et al., 2012). The process of rumen development significantly affects bacterial diversity as revealed by a considerable compositional heterogeneity of rumen microbiota observed in pre-ruminant calves during early development. It is mainly attributed to substantial changes in the anatomy of the rumen wall and subsequent alterations in shifts in physiological function and metabolism (Li et al., 2012). The intimate cross-talk between rumen microbiome, its metabolites, diet, and the host is responsible for successive changes that occur during rumen development. For example, VFA produced by microbes ultimately determine the size and shape of rumen ruminal papilla. These ruminal papillary structures affect microbial colonization as they provide niche environments for certain rumen microbes (Rieu et al., 1990). Cellulolytic bacteria, sulfur-reducing bacteria, and hydrogenotrophic species (methanogens) constitute major functional groups of rumen microflora that become established during the first few days of life (Anderson et al., 1987; Morvan et al., 1996).

Despite the presence of a core microbiome, there are also a large number of microbial taxa, which varies among hosts explaining host specificity of rumen microbiota. Such host specificity of microbes for a specific host does not appear to be restricted to rumen bacteria only but also has been seen with other microbial communities including archaea and the protozoa (Zhou et al., 2012). Rumen microbiomes have an impact on host metabolism and production performance owing to a positive association of rumen microbiome with the host metabolism (Xue et al., 2020). Recent studies on the bovine rumen demonstrated that the rumen microbiome is influenced by host genetic factors (Wallace et al., 2019). That is why selective breeding can be the best alternative to unlock a host's genetic potential and to induce desired changes in the rumen microbiota compared to rumen transplantation, and probiotics (Difford et al., 2018). Recently, Abbas et al. (2020) revealed that there is a direct relationship between rumen microbiota and host genotype that leads to selective absorption of volatile fatty acids, which can increase energy availability to the host animal. The interaction of the host genome with rumen bacterial composition opens up a new horizon for using genomic selection to sustainably improve animal health and productivity. This needs further investigation to devise suitable strategies for targeted manipulation of the rumen microbiome.

Very little information is available regarding host-specific microbial taxa and their physiological and metabolic effects. Moreover, mechanistic insights regarding the role of host genetics to establish a specific niche for particular microbial taxa are lacking. Although, recent high throughput sequencing and analytical advances have piled up a huge dataset regarding microbiota composition and diversity which has surpassed our capacity to elucidate the physiological and ecological roles of individual microbial taxa, especially those that substantially affect the performance of ruminants. That is why the most challenging task is to dissect the physiological roles of microbes to exploit them for manipulation of gut development and microbial colonization to subsequently enhance the productivity of ruminants.

## 6 Future implications

The gut microbiota is a crucial consideration for optimizing better health and the performance of neonatal ruminants. This is mainly because dietary and management practices greatly influence gut microbiota subsequently leading to an alteration in the efficiency of nutrient utilization and the immune response. Studies have clearly demonstrated early life as a window of opportunity in which t manipulation of gut microbiota to mediate the immune response and metabolism of young ones. Dietary interventions in the early days of life have shown substantial effects on gut development and microbial colonization as colostrum feeding and supplementation of pro- and prebiotics has shown desirable effects on calf health and growth. The weaning transition period is also very crucial for the long term and persistent effects on rumen microbiota establishment. Therefore, suitable dietary strategies (like starter feed, supplementation of essential oils and other additives) are required to desirably affect successful rumen development and colonization of beneficial microbes to sustainably improve the performance of neonatal ruminants. However, further studies are required to better understand the mechanism of action of dietary interventions on gut development and microbial colonization. Moreover, a role of host genetics shaping microbial populations and a better understanding of molecular mechanisms through which microbes influence the host physiology during the first days of life is crucial for the identification of pathways and key factors associated with host–microbe interaction.

## Conflict of interest

We declare that we have no financial and personal relationships with other people or organizations that might inappropriately influence our work, and there is no professional or other personal interest of any nature or kind in any product, service and/or company that could be construed as influencing the content of this paper.
